# The Ubiquitin Conjugating Enzyme: An Important Ubiquitin Transfer Platform in Ubiquitin-Proteasome System

**DOI:** 10.3390/ijms21082894

**Published:** 2020-04-21

**Authors:** Weigang Liu, Xun Tang, Xuehong Qi, Xue Fu, Shantwana Ghimire, Rui Ma, Shigui Li, Ning Zhang, Huaijun Si

**Affiliations:** 1 College of Agronomy, Gansu Agricultural University, Lanzhou 730070, China; wgliu@st.gsau.edu.cn (W.L.); shantwana.ghimire@gmail.com (S.G.); mr447233254@126.com (R.M.); lisg@st.gsau.edu.cn (S.L.); 2 Gansu Provincial Key Laboratory of Aridland Crop Science, Gansu Agricultural University, Lanzhou 730070, China; tangxun@gsau.edu.cn (X.T.); xhqi@st.gsau.edu.cn (X.Q.); fox1315@yeah.net (X.F.); 3 College of Life Science and Technology, Gansu Agricultural University, Lanzhou 730070, China; ningzh@gsau.edu.cn

**Keywords:** ubiquitin, ubiquitin-conjugating enzyme (E2), ubiquitin-proteasome system, E2 functions

## Abstract

Owing to a sessile lifestyle in nature, plants are routinely faced with diverse hostile environments such as various abiotic and biotic stresses, which lead to accumulation of free radicals in cells, cell damage, protein denaturation, etc., causing adverse effects to cells. During the evolution process, plants formed defense systems composed of numerous complex gene regulatory networks and signal transduction pathways to regulate and maintain the cell homeostasis. Among them, ubiquitin-proteasome pathway (UPP) is the most versatile cellular signal system as well as a powerful mechanism for regulating many aspects of the cell physiology because it removes most of the abnormal and short-lived peptides and proteins. In this system, the ubiquitin-conjugating enzyme (E2) plays a critical role in transporting ubiquitin from the ubiquitin-activating enzyme (E1) to the ubiquitin-ligase enzyme (E3) and substrate. Nevertheless, the comprehensive study regarding the role of E2 enzymes in plants remains unexplored. In this review, the ubiquitination process and the regulatory role that E2 enzymes play in plants are primarily discussed, with the focus particularly put on E2′s regulation of biological functions of the cell.

## 1. Introduction

Researchers have successfully extracted a small molecular protein (initially called APF-1 and later renamed as “Ubiquitin”) from reticulocytes, and this protein is a key factor in ATP-dependent substrate proteolysis and is universally present in many organisms [1,2,3,4]. Based on these experiments and later research findings, the Nobel Prize in Chemistry was awarded to Aaron Ciechanover, Avram Hershko and Irwin Rose in 2004, for their outstanding contributions to the discovery of ubiquitin-mediated protein degradation [5]. From then onwards, an increasing number of researchers devoted themselves to the ubiquitin-proteasome system (UPS). As a result, the cascade reaction process of this has been basically elucidated at present. Previous studies have shown that the ubiquitin-proteasome system regulates various biological functions of the plant as diverse as hormone responses (including auxin, jasmonic acid, gibberellins, ethylene, and abscisic acid) [6,7,8,9,10,11], abiotic stress response [12,13,14,15], plant growth and development [16,17], circadian rhythm [18], and plant immunity [19,20]. However, the nonproteolytic functions and mechanisms of most key enzymes (over 1600 loci in *Arabidopsis thaliana* genome) which are core components of the UPS (Ubiquitin-proteasome system) are still unclear, especially E3s (more than 1400 potential E3s are encoded in *A. thaliana* genome) and E2s (37 E2s are encoded in *A. thaliana* genome) [8,21]. Therefore, it will be an important task for future research to uncover the role of these enzymes.

## 2. Ubiquitin

Ubiquitin, a small protein of 76 amino acids acting as a tag in post-translational modification of proteins, is absolutely conserved in vertebrates and higher plants, and only two or three amino acid residues are different even among animals, plants, and fungi [22]. The high conservatism renders ubiquitin interchangeable and universal in different species [23]. Ubiquitin is a β-grasp fold protein consisting of a 3.5-turn α-helix, a short 3_10_ helix against a five-strand mixed β-sheet and seven reverse turns [24]. Ubiquitin possesses two hydrophobic surfaces. One surface consists of Ile44, Leu8, Val70, and His68 [25], and the other focuses on Ile36 that can be recognized by HECT (Homology to E6-AP C terminus) E3s, DUBs (Deubiquitinating enzymes), and UBDs (Ubiquitin binding domains) [26,27,28]. Ubiquitin contains seven lysine residues, including Lys-6, Lys-11, Lys-27, Lys-29, Lys-33, Lys-48, Lys-63 (K6, K11, K27, K29, K33, K48, and K63, respectively) [29,30]. Six of the seven Lys linkages (with a preference for Lys-48>Lys-63>Lys-11>>>Lys-33/Lys-29/Lys-6) were detected in *A. thaliana* [31,32]. Any one of the seven lysine residues or *N*-terminal Met1 residues in ubiquitin is likely to be involved in ubiquitin chain formation in vivo, and different ubiquitin linkages lead to the formation of different types of ubiquitin chains and induce diverse molecular signals in the cell [29,33,34,35,36]. Different ways of linking ubiquitin chains and substrates result in distinct fates of the substrates. It is well-known that K48-linked chains are mediators signaling proteasomal degradation and have diverse functions in the cell. For example, they respond to the abiotic/biotic environments [21,37,38]. Some researchers found that K11-linked chains also served as targeting signal for proteasomal degradation in vitro and in vivo [39]. Besides, it was observed in some studies that K63-linked chains not only served as a targeting signal for protein degradation [40], but also had non-degradative functions, such as DNA repair and NF-kb signaling, activation of protein kinases, and protein trafficking [37,41]. Lys-33-linked chains might have nothing to do with protein degradation because they did not increase if the proteasome was inhibited [37], However, Lys-33-linked chains were reported to have nonproteolytic functions, such as, negatively regulating the activity of AMPK (AMP-activated protein kinase) [42]. However, the biological functions of ubiquitin-ubiquitin linkages composed of K6, K27, and K29 are less understood. There is very limited information available on mechanisms and biological functions of different polyubiquitin linkages in prior studies, so researchers must focus on the mechanisms and action processes of polyubiquitination in the future.

In comparison, the small ubiquitin-related modifier (SUMO) which contains approximately 100–115 amino acids is similar to ubiquitin. The SUMO emerges as an influential class of conjugation of protein tags or a modifier to target proteins, and evidence proves that it plays a regulatory role in transcriptional control, subcellular trafficking, and cell cycle [43,44,45]. SUMOylation is driven by three components, which are heterodimeric SUMO-activating enzyme (E1), SUMO-conjugating enzyme (E2), and the substrate recognition factor (E3) [46,47]. SUMOylation is a reversible process, and the SUMO can be released from protein conjugates by SUMO proteases for SUMO recycling [45,48].

## 3. The Ubiquitin Enzymes and Ubiquitination Reactions

The ubiquitin-proteasome system/ubiquitin-proteasome pathway is a prevalent protein turnover system that can degrade or modify proteins in eukaryotic cells. The system can modulate numerous cellular processes, and is almost involved in all aspects of growth and development, so the modification of ubiquitination may be more important than modification of phosphorylation. The UPS consists of ubiquitin (Ub), ubiquitin-activating enzyme (E1), ubiquitin-conjugating enzyme (E2), ubiquitin-ligase enzymes (E3), 26S proteasome, DUBs and target proteins [49,50,51], and it involves two consecutive processes. A chain of ubiquitin molecules selects and labels abnormal and short-lived peptides proteins and substrates at first, then the targets are broken down by the 26S proteasome, and finally the ubiquitin molecules are recycled for a further ubiquitination cycle.

**Figure 1:**
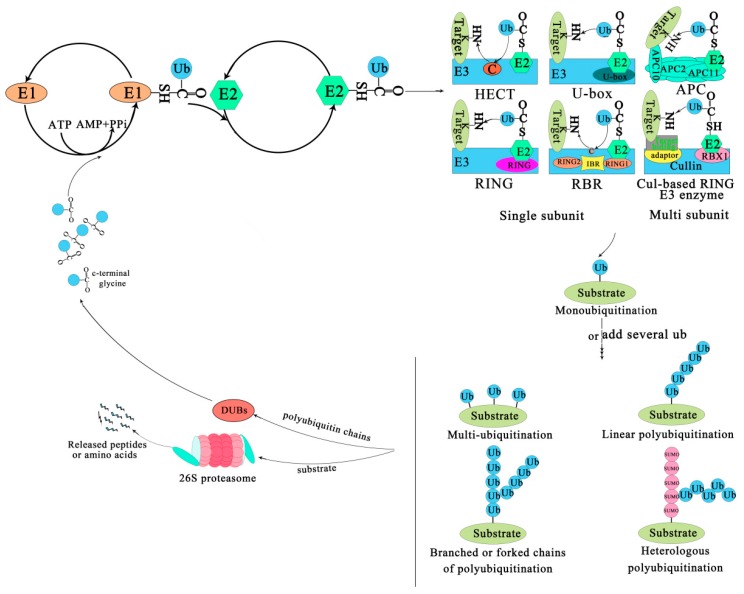
The ubiquitin-proteasome system. The process of ubiquitination from activation to attachment to the substrate is catalyzed by three major enzymes. The substrates labeled by ubiquitin are degraded by the 26S proteasome or play a non-degradative role in other processes. Abbreviations: APC, Anaphase-promoting complex; DUBs, Deubiquitinating enzymes; E1, Ubiquitin-activating enzyme; E2, Ubiquitin-conjugating enzyme; E3, Ubiquitin-ligase enzyme; Cul-based, Cullin-RING box1-Ligase; HECT, Homology to E6-AP C Terminus; Ub, Ubiqitin; SUMO, Small ubiquitin-related modifier; RBX1, RING-Box 1; RING, Really interesting new gene; RBR, RING1-IBR(cysteine/histidine rich region)-RING2.

The covalent attachment of ubiquitin to specific target proteins is mainly accomplished by stepwise enzymatic cascade reactions, and ubiquitin is attached to the substrates via the concerted action of ubiquitin-activating enzyme (E1), ubiquitin-conjugating enzyme (E2), and ubiquitin ligase (E3) [52,53,54]. The attachment of ubiquitin or ubiquitin chains to the substrate is a successive process (Figure 1). First, an E1-ubiquitin thioester bond is formed between the *C*-terminal Gly carboxyl group of ubiquitin and the active site Cys of the E1 enzyme by an ATP-dependent reaction. Then, the E1 transfers the activated ubiquitin to the Cys residue of the E2 enzyme to form an E2-ubiquitin thioester-linked intermediate by transesterification. Eventually the E2 transfers the ubiquitin to the substrate protein by E3 [55]. Ubiquitin is conjugated to the target protein through an isopeptide bond between its *C*-terminal glycine (Gly76) and the ε-amino group of a lysine residue [56]. There are three typical ways of linking the ubiquitin with the substrate. The first is called mono-ubiquitination, which refers to the modification of one site of the modification of a substrate by a single ubiquitin molecule. The second is multi-mono-ubiquitination, which means adding several ubiquitin molecules repetitively to distinct sites (multi-mono-ubiquitination). The third is called polyubiquitination (including linear polyubiquitination and branched polyubiquitination), in which ubiquitin molecules are added to the same site (polyubiquitination, including linear polyubiquitination and branched polyubiquitination) of a substrate. In the second and third ways of linking, the previously attached ubiquitin serves as the “acceptor” of subsequently added ubiquitin [51,54,57,58]. Of course, polyubiquitin chains linked by the same Lys are homogeneous, while those linked by different Lys are heterogeneous or mixed ones [59,60]. Subsequently, the substrate complex tagged by the ubiquitin is degraded by the 26S proteasome or executes nonproteolytic functions, such as some specific biological functions [41]. In most cases, the modified protein (polyubiquitination) is recognized and degraded by the 26S proteasome, and the ubiquitin or ubiquitin chain hydrolyzed and freed by deubiquitinating enzymes (DUBs) for further conjugation cycles after being removed from the substrate protein [61] (Figure 1). Nevertheless, the multi-mono-ubiquitination and mono-ubiquitination that can recruit binding partners, inhibit interactions, alter protein localization, or regulate protein activities [51,62] are necessary for different stages of the secretory/endocytic pathway when certain cargo proteins enter vesicles [51].

The family of E3 enzymes is large and diverse. Over 1400 E3s have been found in *A. thaliana* and they can be divided into U-box domain, RING (Really interesting new gene), HECT (Homologous to the E6AP carboxyl terminus) and Cullin–RING ligases (CRLs) [63,64]. CRLs are subdivided into SKP1-Cullin-F-box (SCF) type, broad complex/tramtrack/bric-a-brac (BTB) type, DDB1-binding/WD-40 domain-containing proteins (DWD) type, and anaphase-promoting complex (APC) [21]. An E3 is a protein or protein complex that conjugates to an E2 and a substrate. It can interact with the substrate directly or through substrate recognition modules of seven types of E3 ligases via the adaptor and target binding proteins (Figure 1). For example, the HECT enzyme transfers ubiquitin from an E2 to its Cys residues before it binds to the target protein NH2 conjugate [65,66]. The RING/U-box enzyme acts as a scaffold for the aggregation of an E2 and a substrate, directly transferring ubiquitin from an E2 to a substrate; Cul-based RING enzyme transfers ubiquitin from E2 to the substrate that binds with an accessory factor.

## 4. The Family of E2 Enzymes

The E2 enzyme is a key transfer site of ubiquitination, which interacts with E1 and E3 respectively. There are two puzzling questions about the ubiquitin-proteasome system. One is why E2 enzymes are present in all eukaryotes and the other is why we do not directly transfer ubiquitin from E1s to E3s. Some researchers believed that an E2 enzyme could combine with different types of E3s that recognized and selected distinct targets, and thereby played a decisive role in determining whether the labeled protein would be degraded or involved in nonproteolytic processes. In other words, E3s mainly select the substrate, while E2s determine the fate of the substrate [67]. Another possible and interesting consideration is that E2s promote ubiquitination independent of an E3 ligase, such as UBC22 [68,69].

Each E2 contains a core UBC domain composed of about 150–200 amino acid residues, in which the cysteine is an active site for the formation of thioester linkages [54,70]. The UBC domain has a structural conservation rate of 35% in different species [71], consisting mainly of four α-helices, an anti-parallel β-sheet and a short 3_10_ helix [72,73,74]. Almost all UBC domains contain a conserved His-Pro-Asn tripeptide (HPN) located at about the tenth residue at the N-terminal of the Cys residue [2,75]. Aspartic acid in HPN catalyzes the formation of the isopeptide bond, histidine is essential for the structure, and proline promotes stable transition of these amino acid residues [75,76]. According to the location of the additional fragment in the UBC domain, the family of E2 enzymes can be categorized into four classes. Class I is present only in the UBC domain; class II is present in both *N*-terminal and UBC domain; class III is present in both *C*-terminal and UBC domain; and class IV is present in *N*-terminal, *C*-terminal and UBC domain [67]. E2 enzymes belong to the polygenic family, and the number of members varies from a dozen to dozens in different species. 20 E2 enzymes have been found in *Caenorhabditis elegans* [77], 40 in *A. thaliana* [68], 14 in *Saccharomyces cerevisiae* [78], 37 in human [67], 48 in rice [79], 75 in maize [80], 72 in banana [81], 43 in *Vitis vinifera* [82], 34 in *Carica papaya* [83], 59 in tomato [84], 40 in *Dimocarpus longan* Lour. [85], and 57 in potato [86].

**Figure 2:**
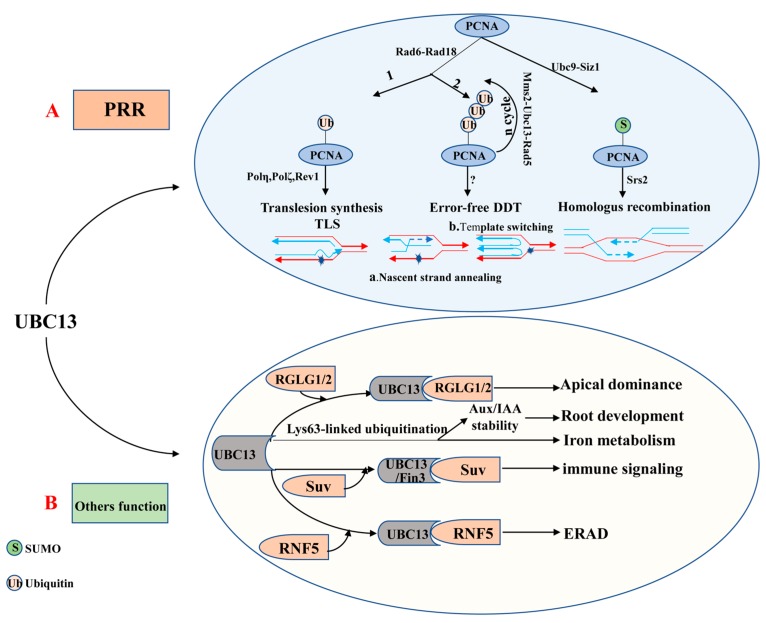
The role of UBC13 in DNA damage responses and UBC13′s other functions. (**A**) UBC13 is involved in post-replication repair (PRR). (**B**) UBC13 participates in different biological processes by mediating Lys63-linked ubiquitination. Abbreviations: Aux/IAA, Auxin/indole-3-acetic acid; DDT, DNA damage tolerance; ERAD, ER-associated degradation; Mms2, A Ubc E2 variant; PRR, Postreplication repair; PCNA, Proliferating cell nuclear antigen; RGLG1/2, An E3 ligase; RNF5, RING finger protein 5; Rad5, An E3 enzyme; Rad6, An E2 enzyme; Rad18, An E3 enzyme; Suv, Fni3 (Fen-interacting protein, Fen is the host protein kinase) cofactor *S. lycoperiscum* Uev; Siz1, An E3 ligase; Srs2, A helicase; TLS, Translesion DNA synthesis; Ubc9, An E2 enzyme.

The ubiquitin E2 variant (UEV) is similar to the E2 enzyme in both sequence and structure, but it cannot form a thioester linkage with ubiquitin because of the lack of the catalytic cysteine residue [87,88]. The first UEV gene was initially defined as MMS2, which was obtained from budding yeast cells and associated with the error-free DNA-damage tolerance (DDT) pathway [87,89]. Although UEVs cannot conjugate with ubiquitin, UEV proteins form a stable heterodimer with E2s to facilitate the ubiquitination of a target protein [68,90,91]. It is clear that UBC13 usually combines with a heterologous UEV to form a complex which plays a critical role in DNA repair. The heterodimer-coupled E3 Rad5, for instance, can functionally remedy the corresponding ubc13 mutants’ defect in PRR (post-replication repair, also known as error-free DNA-damage tolerance) [87,92,93] (Figure 2A). The newly discovered expression of *OsUEV1s* is also capable of functionally rescuing the yeast mms2 mutant from death caused by DNA-damaging agents [94]. Much more importantly, the stable heterodimer formed by UBC13 and a UEV (from yeast or mammalian cells) is essential for assembly of Lys63-linked polyubiquitin chains [91,93,95]. 4 *AtUev1s*, 4 *OsUev1s*, and 3 *BdUev1s* have been found in *A. thaliana*, rice and *Brachypodium distachyon,* respectively [94,96,97]. COP10 is a type of UEV, which is essential to protein degradation [98]. Besides, COP10 plays a vital role in photo-morphogenesis and response against UV-B [90]. It remains unknown whether UEVs have other independent biological functions or play a role in regulating biological processes together with E2 or E3 enzymes. Therefore, this will be the direction for research in this field shortly.

## 5. Functions of E2 Enzymes

The E2 enzymes are one of the key parts of the ubiquitin-proteasome system, having special significance and functions (Table 1).

### 5.1. Abiotic Stress Response

*VrUBC1* is a ubiquitin-conjugating enzyme gene from mung bean, and responds to dehydration, high salinity and abscisic acid (ABA) treatment. *VrUBC1*-overexpressed plants are more sensitive to abscisic acid-mediated stomatal closure and have more tolerance to drought stress, indicating that *VrUBC1* is a positive regulator under osmotic stress in *A. thaliana* [99]. *OgUBC1* is a ubiquitin-conjugating enzyme gene from wild rice, triggered by salicylic acid in leaves [100]. Tobacco with overexpression of *NtUBC1* exhibits increased resistance to cadmium (Cd) compared to WT [101]. A significantly increased expression of *LeUBC1* under the conditions of heat shock and cadmium chloride (CdCl2) indicates that *LeUBC1* can respond to these two stresses [102]. *AhUBC2* is a ubiquitin-conjugating enzyme gene in peanuts, which can respond to PEG6000, high salinity, abscisic acid (ABA) and low temperature. Higher levels of *P5CS1*, *RD29A*, and K*IN1* under a normal condition, and a higher level of proline under both soil-drought stress and control conditions are found in *35S:AhUBC2* transgenic plants, suggesting that the constitutive expression of *AhUBC2* improves water-stress tolerance by activating an ABA-independent signaling pathway [103]. The *GmUBC2* from soybean is the yeast homolog *Rad6*, capable of improving salt and drought tolerance, and it is involved in the regulation of ion homeostasis, osmolyte synthesis, and oxidative stress responses in *A. thaliana* [104]. Similarly, the increased expression of *CmUBC* in transcript level in melon plants under drought and salinity stresses indicates that *CmUBC* can respond to physiological water stress [105]. The Rad6 gene of *Hevea brasiliensis* shares a similarity of over 96% with OsRad6 in rice and *AtUBC2* in *A. thaliana*, and its expression can be markedly induced by the latex stimulator ethephon (ET) and methyl jasmonate (MeJA) [106]. *RCE1* from *A. thaliana* is homologous with the human *UBC12* gene, and its conjugation with the ubiquitin-like protein RUB1 that combines with AtCUL1 affects the functions of SCF E3s and thus influences the auxin response [107,108]. A study showed stable and high expression of *OsUBC13* under different biotic and abiotic stresses, suggesting *OsUBC13* as a housekeeping gene [109]. Recent research has shown that UBC13 is a key regulator of *A. thaliana* response to low temperature [110] (Figure 2B). UBC18 can interact with ethylene response factor 1 (ERF1), and the differences in abundance of *ERF1* between *UBC18* mutants and *UBC18* overexpression lines are caused by ubiquitination. Down-regulation of *UBC18* leads to increased *ERF1* and proline accumulation. Besides, *UBC18* negatively regulates responses to drought and salt stresses [111]. AtUBC32, AtUBC33, and AtUBC34 can interact with PUB19, a negative regulator of the drought stress response. Repression of *AtUBC32*, *AtUBC33*, and *AtUBC34* expression in both single and triple mutant lines can promote abscisic acid-mediated stomatal closure and improve tolerance to drought stress, indicating that *AtUBC32*, *AtUBC33*, and *AtUBC34* play a negative regulatory role in drought stress through mediation of the abscisic acid [112]. *AtUBC32*, *AtUBC33*, and *AtUBC34* belong to group XIV UBCs, and UBC32-GFP is located in ER membranes [113]. UBC32 can interact with the RING ligase DOA10B (At4g32670) which is remarkably similar to yeast Doa10 (Degradation of Alpha2). *UBC32* mutants are extremely sensitive to paraquat treatment compared with overexpression plants, suggesting that UBC32 is an essential ERAD component and plays an important part in the response to oxidative stress and salt stress [114]. Although changes in expression of UBC genes in *A. thaliana* and rice are not induced by cold stress treatment [103], some reports have found that the UBCs gene in other species have responses to cold and heat stresses [80,82]. *UBC24* (pho2) is regulated by microRNA399 (miR399) to control inorganic phosphate (Pi) homeostasis and Pi translocation and remobilization [115,116,117,118].

### 5.2. Plant Immune Responses

Fni3, Lys-63-linked ubiquitination, is a homolog of the UBC13-type ubiquitin-conjugating enzyme. A decreased expression *of Fni3* or the ubiquitin E2 variant *Suv* can affect Fen-mediated immunity and cell death caused by several other resistance (R) proteins and their cognate effectors (Figure 2B). This study suggests that *Fni3/Sl-Ubc13-2* and *Suv* positively regulate plant immunity [128]. By knocking down the Group III E2 genes in *Nicotiana benthamiana* via VIGS (gene silencing), it is found that these genes are necessary for plant development, immunity-associated ROS production, and suppression of immunity-associated PCD (programmed cell death) by *AvrPtoB* [134,135]. Besides, knocking down the expression of *TaU4* gene which encodes a ubiquitin-conjugating enzyme 4 in *Triticum aestivum* by VIGS can slow down the development of disease symptoms and reduce *Septoria sporulation* (*Mycosphaerella graminicola,* commonly known as *Septoria*), indicating that *TaU4* is a negative regulator for defense against the phytopathogen *Septoria* in wheat [123]. The group III E2 genes act together with SlPUB13 on the ubiquitination of FLS2 in vitro and regulate FLS2-mediated immune signaling [136]. Heterologous expression of the *OgUBC1* in *A. thaliana* defends plants from UV-B-mediated cell damage and enables them to resist *Botrytis cinerea* [100].

### 5.3. Plant Growth and Development UBC13 Catalyzes Non-Canonical Lys63-Linked Ubiquitin Chains

*A. thaliana* carrying UBC13 mutants can display strong phenotypes and is unable to grow root hairs in case of iron deficiency. Especially, *A. thaliana* containing UBC13A and UBC13B mutants have a significantly reduced root-hair density. Therefore, it is concluded that UBC13A/B is involved in epidermal cell differentiation and iron deficiency responses [117,137]. UBC13A/B mutants of *A. thaliana* can influence primary and lateral roots [137,138] (Figure 2B). A VIGS analysis showed that SlUBC32 and SlUBC41 were implicated in the regulation of fruit ripening [139]. UBC22 is the sole member of subfamily X, and can catalyze the assembly of ubiquitin chains in the absence of E3 in vitro [68]. Silique length and the seed numbers are significantly decreased and nearly 90% of the ovules are aborted after knocking out UBC22 gene mutants in *A. thaliana*, indicating that UBC22 is required for the development of female gametophytes [140]. The RING finger protein TaRF1 can interact with TaUBC28, which potentially plays a role in spike development of wheat [131]. UBC21 is better known as PEROXIN 4 (PEX4). Pex4/ubc21 mutants have reduced root elongation because of their resistance to indole butyric acid (IBA), while the major auxin indoleacetic acid (IAA) processed from IBA can significantly promote the root growth of pex4 mutants [129]. There are two E3 homologs (HUB1 and HUB2) and three RAD6 homologs (UBC1, UBC2, and UBC3) in *A. thaliana* [120]. As reported by researchers previously, HUB1 and HUB2 were involved in regulating seed dormancy, leaf and root growth [141,142]. The ubc1 and ubc2 mutants displayed an early-flowering phenotype, and they worked together with HUB1 and HUB2 to control the flowering time of *A. thaliana* [141,143,144]. Similar studies involving AtUBC1/AtUBC2 and HUB1/HUB2 also have shown that these four genes mediate the H2B ubiquitination, thereby activating FLC expression and repressing flowering of the plant [119].

### 5.4. Error-Free DNA-Damage Tolerance and Repair

To date, UBC13 is the only E2 known to be able to catalyze K63-linked polyubiquitin chain assembly in eukaryotes [93,145,146]. In most cases, the K63-linked polyubiquitin chain assembled combines with a UEV (e.g., MMS2) as a cofactor [93,95,147]. Evidence is accumulating to suggest that the UBC13-MMS2 complex is involved in DTT (also known as DNA post-replication repair) and DNA repair [147,148,149,150] (Figure 2A). DNA radiation repair pathways are mainly divided into nucleotide excision repair, homologous recombination repair, and the damage tolerance pathway initially. Interestingly, the damage tolerance pathway is different from the other two major pathways as it does not remove or repair the original lesion but bypass the lesion [151]. It is now clear that PRR includes two branches, namely the error-prone (mutagenesis) branch and the error-free branch [87,152,153]. The error-prone branch is also called DNA translation synthesis (TLS), which is mediated by non-essential DNA polymerases including Polη (Rev3+Rev7), Polζ, and Rev1. By contrast, the error-free branch is mediated by the current model Ubc13-Mms2 [92,154,155]. These two independent sub-pathways typically rely on RAD6 and RAD18 [149,156], which are recruited to the stalled replication fork by RPA to facilitate mono-ubiquitination of the proliferating cell nuclear antigen (PCNA). Rad6 from *Saccharomyces cerevisiae* is a ubiquitin-conjugating enzyme (E2), which is proven to play a role in DNA repair, damage-induced mutagenesis, and sporulation [157,158]. In fact, for the former, PCNA encoded by Pol30 at the K164 is mono-ubiquitinated by the Rad6-Rad18 to promote TLS, while for the latter, mono-ubiquitin-POL30 is further poly-ubiquitinated by the Mms2-Ubc13-Rad5 complex to facilitate error-free damage tolerance [159,160]. Besides, the PCNA can also be modified by the SUMO at K164 under the catalysis of the E2-E3 complex Siz1-Ubc9 [159,161]. The DNA helicase Srs2 enters the stalled replication forks under the mediation effect of the SUMOylated PCNA, suppressing the redundant and harmful homologous recombination [162,163,164].

## 6. Concluding Remarks and Future Perspectives

E2 enzymes are the intersection between E1 and E3 enzymes and determine the ubiquitination of specific target proteins by tagging different types of E3 enzymes. This review elaborates on some of the reported functions of E2 enzymes. Since the direct interaction between E3 enzymes and substrates results in the substrate ubiquitination, previous studies mainly focused on the mechanisms and functions of E3 enzymes. E3-mediated substrate degradation has always been the concern of research, but E2 enzymes that play a crucial role in this mechanism have not gotten much attention. The present model of ubiquitination only expounds the process of Ub transferring. The findings on the driving mechanism of E2 enzymes recognizing diverse E3 and specific substrates are very limited. Moreover, it is wondered whether E2 enzymes have other biological functions independent of the ubiquitin-proteasome system, such as regulating other signaling pathways to regulate the expression of downstream genes through signaling pathways. Much more importantly, only a few studies have investigated the role of E2 enzymes, especially in plants. Therefore, future research should focus on clarifying above important issues and explore new concerned research directions.

## Figures and Tables

**Table 1:** Ubiquitin-conjugating enzymes with different known functions ^a^.

E2 Enzyme	Species	General Functions Description	Reference
*NtUBC1*	*Nicotiana tabacum*	Cadmium tolerance	[101]
*LeUBC1*	*Lycopersicon esculentum*	Heat shock, cadmium chloride	[102]
*VrUBC1*	*Mung Bean*	Salinity/ABA (abscisic acid)/drought stress	[99]
*AtUBC1*	*Arabidopsis thaliana*	Regulate flowering time	[119]
*OgUBC1*	Rice	Salicylic acid/resistant to *Botrytis cinerea*l and UV-B radiation	[100]
*Ubc2/Rad6p*	Yeast and plants	Virus replication/DNA repair/ERAD (ER-associated degradation)/histone ubiquitination/Flowering time/salt and drought-tolerance	[67,106,120,121,122]
*AhUBC2*	Dehydrated peanut plants	Water-stress tolerance	[103]
*GmUBC2*	Soybean	Salt and drought tolerance/ion homeostasis	[104]
*TaU4*	wheat	Defense against *Septoria*	[123]
*Ubc7*	-	ERAD	[124]
*OsUBC13*	rice	Biotic and abiotic stresses/error-free PRR (postreplication repair)	[109]
*AtUBC13*	*A.thalianaArabidopsis thaliana*	Error-free DDT (DNA-damage tolerance)	[125,126]
*ShUbc13*	*Sterkiella histriomuscorum*	DNA damage response	[127]
*Fni3 / S1-Ubc13-2*	*Solanum lycopersicum*	Plant immunity	[128]
*RCE1 (UBC12)*	*Arabidopsis thaliana*	Auxin response	[107]
*UBC18*	*Arabidopsis thaliana*	Drought and salt stress responses	[111]
*UBC21/PEX4*	*Arabidopsis thaliana*	Root development	[129]
*UBC22*	*-*	Strong E3 (Ubiquitin-ligase enzyme) independentactivity to form ubiquitin chains	[68,130]
*UBC24/PHO2*	*Arabidopsis thaliana*	Phosphate (Pi) homeostasis	[116]
*TaUBC28*	wheat	Spike development	[131]
*AtUBC32*	*Arabidopsis thaliana*	Drought/oxidative/salt stress/ERAD	[112,113,132,133]
*AtUBC33*	*Arabidopsis thaliana*	Drought stress	[112]
*AtUBC34*	*Arabidopsis thaliana*	Drought stress	[112]

a. The table includes some functions of the UBC genes that have been discussed in this paper.
